# Risk factor structure of heart failure in patients with cancer after treatment with anticancer agents’ assessment by big data from a Japanese electronic health record

**DOI:** 10.1007/s00380-023-02238-9

**Published:** 2023-01-27

**Authors:** Shoichiro Nohara, Kazuo Ishii, Tatsuhiro Shibata, Hitoshi Obara, Takanobu Miyamoto, Takafumi Ueno, Tatsuyuki Kakuma, Yoshihiro Fukumoto

**Affiliations:** 1 Division of Cardiovascular Medicine, Department of Internal Medicine, Kurume University School of Medicine, 67 Asahimachi, Kurume, 830- 0011 Japan; 2 Biostatistics Center, Kurume University, Kurume, Japan

**Keywords:** Anticancer agents, Heart failure, Epidemiology, Machine learning, Electronic health record

## Abstract

As the prognosis of cancer patients has been improved, comorbidity of heart failure (HF) in cancer survivors is a serious concern, especially in the aged population. This study aimed to examine the risk factors of HF development after treatment by anticancer agents, using a machine learning-based analysis of a massive dataset obtained from the electronic health record (EHR) in Japan. This retrospective, cohort study, using a dataset from 2008 to 2017 in the Diagnosis Procedure Combination (DPC) database in Japan, enrolled 140,327 patients. The structure of risk factors was determined using multivariable analysis and classification and regression tree (CART) algorithm for time-to-event data. The mean follow-up period was 1.55 years. The prevalence of HF after anticancer agent administration were 4.0%. HF was more prevalent in the older than the younger. As the presence of cardiovascular diseases and various risk factors predicted HF, CART analysis of the risk factors revealed that the risk factor structures complicatedly differed among different age groups. The highest risk combination was hypertension, diabetes mellitus, and atrial fibrillation in the group aged  ≤ 64 years, and the presence of ischemic heart disease was a key in both groups aged 65–74 years and 75 ≤ years. The machine learning-based approach was able to develop complicated HF risk structures in cancer patients after anticancer agents in different age population, of which knowledge would be essential for realizing precision medicine to improve the prognosis of cancer patients.

**Supplementary Information:**

The online version contains supplementary material available at 10.1007/s00380-023-02238-9.

## Introduction

Recent improvements in cancer therapy have increased the number of survivors [1, 2]. As these survivors get older, the comorbidity of cardiovascular diseases (CVDs) has become a serious concern, especially given that age is one of the most important risk factors for CVDs. Among CVDs, heart failure (HF) is a serious condition with poor prognosis [3, 4]. Although multi-drug therapies have become the standard for various types of cancer and that molecular-targeted anticancer agents have been introduced in clinical practice, various anticancer agents have been shown to predispose patients to cardiovascular complications, typically in the form of HF [3–5]. Such unfavorable cardiovascular side effects frequently force clinicians and patients to interrupt anticancer therapy. Although a body of knowledge has been developed from a number of clinical studies, most of the previous studies regarding HF during anticancer treatment were cancer-specific, therapeutic agent-specific, or both in selected patients; however, this is far from a realistic clinical situation in which patients have various types of cancer and complex backgrounds as well as the choice of therapeutic interventions [6–9]. The current risk stratification system for cardiotoxicity seems inadequate due to these heterogeneous factors. This limitation is partly due to the fact that hundreds of thousands of patients need to be enrolled in clinical studies to obtain knowledge regarding the relationships among their complex clinical backgrounds, types of cancers, therapeutic interventions, and HF. It has been impractical to conduct such studies because of the large effort and excessive cost. Another obstacle is the difficulty required to handle and process such a large dataset, as was used in this study. However, recent advancements in computing and data science technologies have made it possible to manage big data. To capture a realistic clinical situation, an electronic health record (EHR) provides a unique opportunity to obtain a dataset that reflects the situation in clinical practice.

To this end, we employed a machine learning-based approach to analyze medical big data extracted from a Japanese EHR stored in the Diagnosis Procedure Combination (DPC) reimbursement system. Our procedure may provide a new analytical approach for discovering a structured risk model. The present study aimed to examine the risk of HF development after the treatment by anticancer agents using medical big data.

## Materials and methods

### Study design and data source

This retrospective cohort study used data from the DPC database of patients who were treated by anticancer agents. Although there is no single EHR database that covers the entire population, the DPC system is the largest hospital-based database and covers 83% of acute-care medical institutions in Japan. We obtained the database of Medical Data Vision Co., Ltd. (Tokyo, Japan), which covered 291 acute care hospitals and 129 of the 393 designated cancer hospitals in Japan. The total number of patients included in the Medical Data Vision Co., Ltd. Database was 17.85 million. The DPC database contains dated information for diagnosis using the International Classification of Disease 10th revision (ICD-10) codes, comorbidities at admission and discharge, and complications that occurred during hospitalization. This database includes the records of both outpatients and inpatients. In addition, data were extracted for procedures, such as medications, medical devices, consumables, physiological and laboratory examinations, and in-hospital deaths as well as basic patient information, such as anonymized patient ID, age, sex, height, and weight. We classified the patients into three age groups (≤ 64 years, 65–74 years, and  ≥ 75 years) according to the World Health Organization (WHO) criteria for early-stage (≥ 65 but  ≤ 75 years) and late-stage (≥ 75 years) elderly patients. This study was approved by the Institutional Review Board of Kurume University (approval number 19115). The requirement for informed consent was waived because all the data were anonymized.

### Definition of cancer and HF

**Fig. 1:**
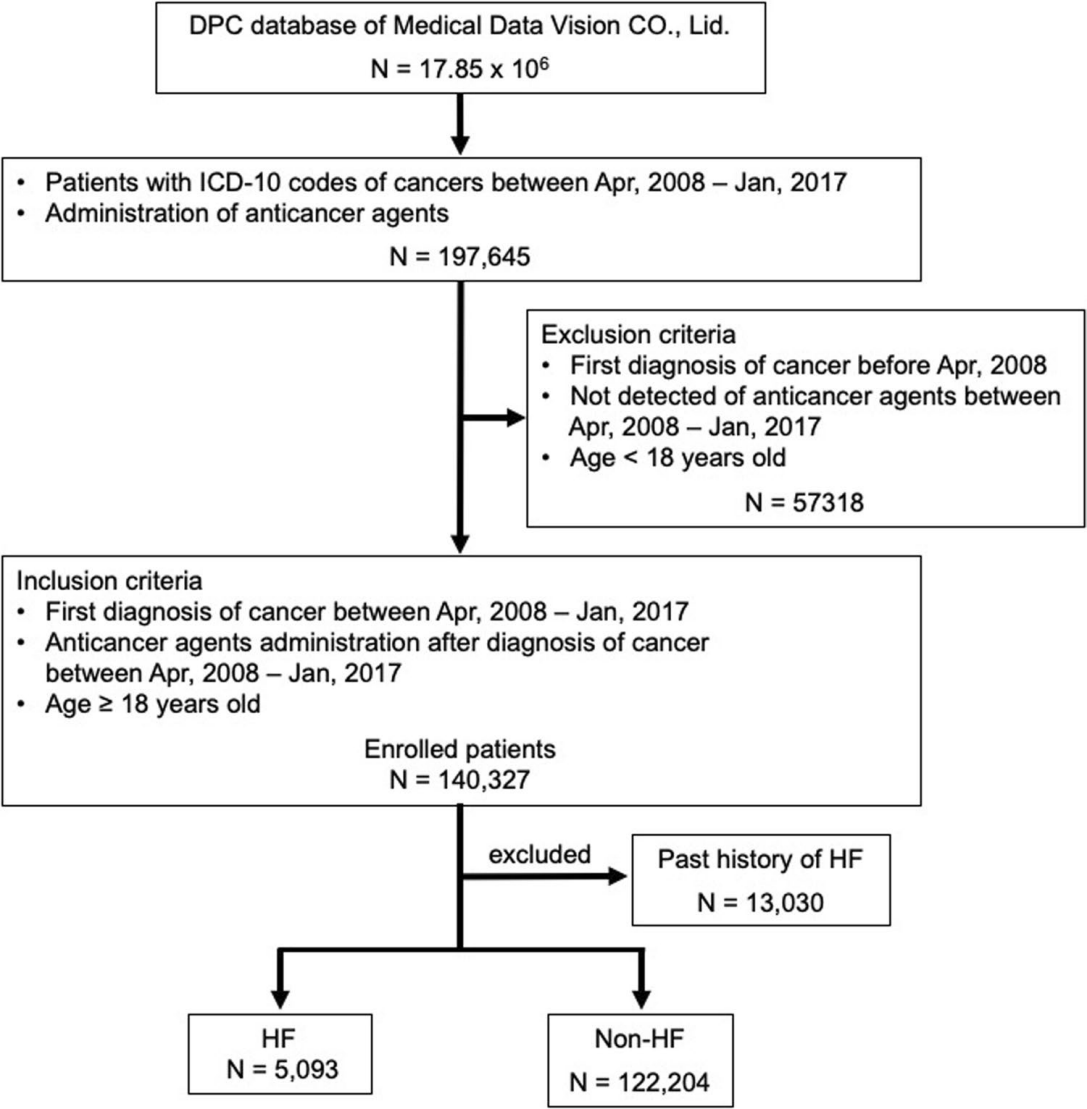
Enrollment of patients treated for cancer and identification of incidences of HF in this study. Patients with cancer were enrolled in this study on the first day of anticancer agent administration. Patients with or without HF diagnosis were selected as depicted

We defined patients with cancer as those with ICD-10 codes C00-C96. As D01-09 are intraepithelial carcinomas, we excluded them from this study because they are unlikely to be clinically targeted for anticancer therapy. We enrolled patients aged 18 years or older at the initial diagnosis of cancer who received anticancer agents between April 2008 and January 2017 (Fig. 1, Supplementary Table 1). The date of enrollment was defined as the time when the anticancer agents were first administered in this study.

We also defined the ICD-10 codes I50.0, I50.1, I50.9, I11.0, and I42.7 as the HF codes (Supplementary Table 2 and 3). In this study, we identified HF patients as those who were labeled with both the HF codes and used one or more of the following therapeutic drugs for HF: angiotensin-converting enzyme inhibitors, angiotensin II receptor blockers, β-blockers, mineralocorticoid receptor antagonists, loop diuretics, tolvaptan, nitroglycerine, carperitide, dobutamine, or phosphodiesterase 3 inhibitors. We excluded patients with HF codes if they were simultaneously coded for acute myocardial infarction or one or more of the other conditions in which clinical signs and symptoms are similar to HF, including cardiopulmonary arrest, acute respiratory distress syndrome, severe pneumonia, pleuritis, or severe renal failure (Supplementary Table 2 and 3). Patients with a history of HF prior to the start of anticancer treatment were also excluded.

For other comorbidities, the diagnosis was made when the patients were coded with ICD-10 codes corresponding to those comorbidities at the time of enrolment.

### Statistical analyses

All data manipulation and extraction were performed with GNU bash (version 4.3.48(1) release) including GNU Awk 4.1.3, GNU grep 2.25, and GNU sed 4.2.2 in the Linux platform (Ubuntu 16.04.6 LTS operating system) on the HPE ML350 Gen9 E5-2699v3 server (36 core CPUs, memory 768 Gb, 9.6 Tb HDD). Specific tables for data analysis were prepared using custom programming in an interactive terminal (GNOME Terminal 3.18.3) or shell scripting with bash. Basic statistics such as means and standard deviations for the summary data were obtained using R version 3.4.4 9 [10]. Classification and regression tree (CART) analysis was performed using the Stata 15 software (Stata Corp LLC, Texas, USA). The Cox proportional hazard model was used to evaluate the risk factors for HF after treatment with anticancer agents. Because cancer death may be considered as a competing risk for the development of HF, the Fine-Gray competing risk model [11] was also used for sensitivity analysis for the Cox proportional hazard model. CART is a non-parametric decision tree learning technique that partitions future space with a set of all possible combinations of a set of risk factors. A partitioned future space consists of an asymmetrical combination of risk factors that provide interpretable patient clinical profiles with various degrees of risk for clinical outcomes. This learning technique has been applied in prospective epidemiological studies and is applicable to our retrospective cohort data [12, 13]. *P*-values less than 0.01 were accepted as statistically significant.

## Results

### Patient enrollment and basic characterization

Of 17.85 million patients in the original DPC database, there were 197,645 inpatients and outpatients who received anticancer agents between April 2008 and January 2017. After applying the inclusion and exclusion criteria (Supplementary Table 1), 140,327 patients were enrolled in the study (Fig. 1). Table 1 shows the baseline characteristics of the patients. Among the patients in the study population, 5,093 (4.0%) were diagnosed with HF after initial administration of anticancer agents (Fig. 1). Upon comparison, patients who experienced HF had a higher average age, and 43.5% of these patients were over 75-years-old. Cardiovascular comorbidities, such as hypertension (HT), ischemic heart disease (IHD), and atrial fibrillation/flutter (AF) and cardiovascular risk factors, such as diabetes mellitus (DM) and dyslipidemia (DL), were also higher in the HF group. This indicates that many patients with cancer had cardiovascular diseases and their risk factors at baseline in this study.

**Table 1:** Baseline characteristics of patients

Parameters	All patients	Non HF groups	HF groups	*P* value
Number	127,297	122,204	5093	
Follow up periods (years)	1.55 [0–8.5]	1.53 [0–8.5]	2.02 [0—8.2]	< 0.01^*2^
Age (years), mean ± SD	68.7 ± 11.7	68.6 ± 11.7	71.3 ± 10.1	< 0.01^*2^
18–64	38,535 (30.3%)	37,411 (30.6%)	1124 (22.1%)	< 0.01^*3^
65–74	44,929 (35.3%)	43,173 (35.3%)	1756 (34.5%)	0.03
≥ 75	43,833 (34.4%)	41,620 (34.1%)	2213 (43.5%)	< 0.01^*3^
Sex (males)	73,689 (57.9%)	70,486 (57.7%)	3203 (62.9%)	< 0.01^*3^
History of smoking^*1^	57,573 (50.1%)	55,079 (50.0%)	2494 (54.5%)	< 0.01^*3^
BMI, mean ± SD	22.6 ± 3.7	22.6 ± 3.7	22.9 ± 3.8	< 0.01^*2^
Hypertension	45,688 (35.9%)	43,185 (35.3%)	2503 (49.1%)	< 0.01^*3^
Ischemic heart disease	13,448 (10.6%)	12,391 (10.1%)	1057 (20.8%)	< 0.01^*3^
Atrial fibrillation/flutter	4898 (3.8%)	4481 (3.7%)	417 (8.2%)	< 0.01^*3^
Ventricular Arrhythmias	2028 (1.6%)	1909 (1.6%)	119 (2.3%)	< 0.01^*3^
Diabetes mellitus	35,270 (27.7%)	33,380 (27.3%)	1890 (37.1%)	< 0.01^*3^
Dyslipidemia	23,280 (18.3%)	21,941 (18.0%)	1339 (26.3%)	< 0.01^*3^
Hyperuricemia	7887 (6.2%)	7318 (6.0%)	569 (11.2%)	< 0.01^*3^
Chronic kidney disease	2831 (2.2%)	2634 (2.2%)	197 (3.9%)	< 0.01^*3^
Cerebrovascular disease	6960 (5.5%)	6574 (5.4%)	386 (7.6%)	< 0.01^*^

^*^1 Data retention rate 90.6%

^*^2 Analysis of variance

^*^3 Chi-squared test

### Risk factors for HF after anticancer treatment

**Fig. 2:**
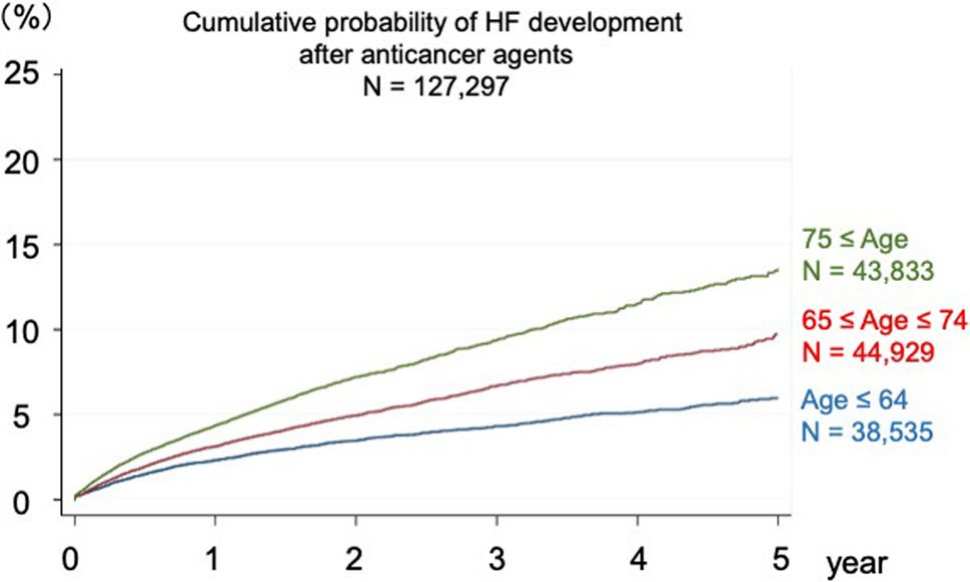
Cumulative probability of HF after anticancer treatment stratified by age. Cumulative probability of HF within 5 years of anticancer therapy in different age groups:  ≤ 64 years, 65–74 years, and  ≥ 75 years

Because the burden of HF is disproportionately distributed among the elderly [14], we divided the subjects into three age groups:  ≤ 64-years-old, 65–74-years-old, and  ≥ 75-years-old. The impact of age on HF after the administration of anticancer agents was assessed using Kaplan-Meier analysis for the three age groups. The HF was more prevalent in the two older groups compared to the youngest age group during the observational period (Fig. 2). Next, we evaluated the risk factors of HF after treatment with anticancer agents. In the Cox’s proportional hazard model after adjusting for sex and comorbidities, HF was more prevalent in the older groups than in the younger group; the hazard ratio was 1.23 (99%CI 1.14–1.33; *P* < 0.01) for the 65–74 years group and 1.67 (99%CI 1.55–1.80, *P* < 0.01) for  ≥ 75 years group compared to the youngest group, respectively (Table 2). Comorbidities of cardiovascular diseases and their risk factors were also significantly associated with HF, except for ventricular arrhythmias and cerebrovascular disease (Table 2). For validation, we analyzed the use of doxorubicin, a known cardiotoxic drug. The hazard ratio was 2.18 (99%CI 1.96–2.42; *P* < 0.001) in the doxorubicin group. This result was compatible with the effect size of already known clinical evidence, indicating the validity of this study.

**Table 2:** Multivariate analysis for HF after treatment with anticancer agents

Variables	Hazard ratio	99% CI		*P* value
Age				
Age ≤ 64 (reference)	1.00	–	–	–
65 ≤ Age ≤ 74	1.23	1.11	1.36	< 0.01
75 ≤ Age	1.67	1.51	1.85	< 0.01
Male	1.05	0.97	1.14	0.08
Obesity	1.20	1.08	1.33	< 0.01
Hypertension	1.28	1.18	1.39	< 0.01
Ischemic heart disease	1.62	1.47	1.79	< 0.01
Atrial fibrillation/flutter	1.81	1.58	2.07	< 0.01
Ventricular Arrhythmias	0.98	0.77	1.24	0.81
Diabetes mellitus	1.29	1.19	1.40	< 0.01
Dyslipidemia	1.11	1.02	1.22	< 0.01
Hyperuricemia	1.49	1.31	1.68	< 0.01
Chronic kidney disease (Stage G3-4)	1.25	1.03	1.51	< 0.01
Cerebrovascular disease	1.01	0.88	1.17	0.81

Adjusted for comorbidity by Cox’s proportional hazards regression model

### Risk factors for HF development

**Fig. 3:**
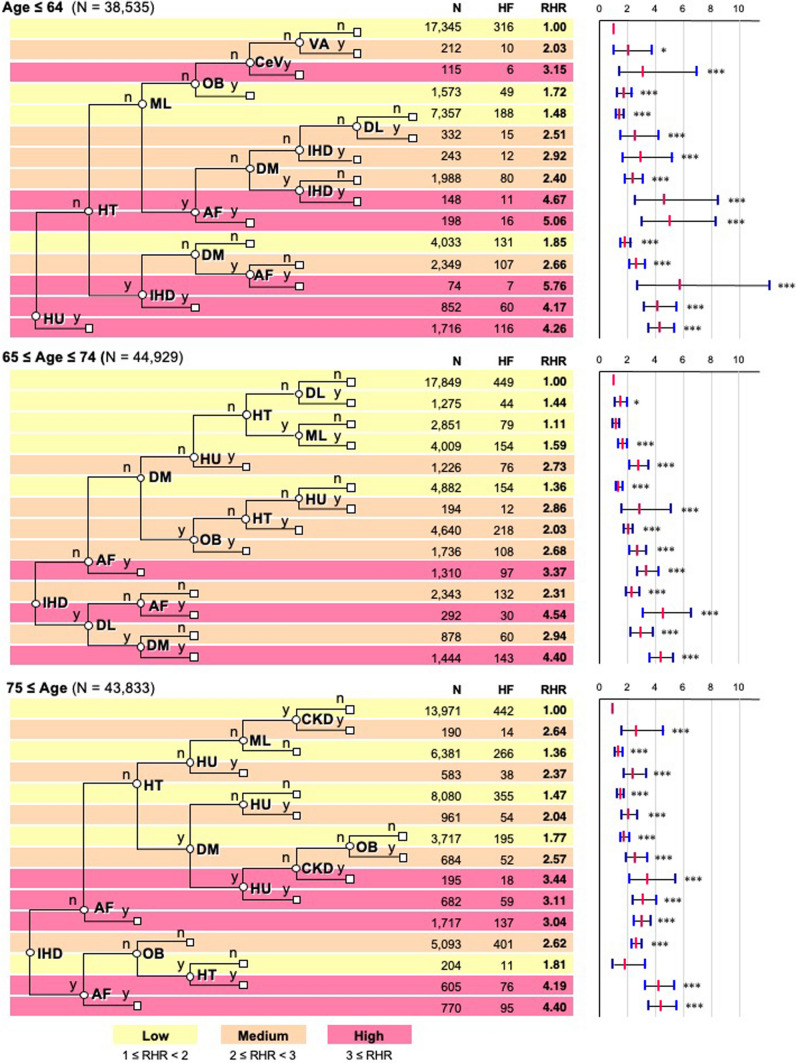
CART analysis of risk factors for HF development. Risk factors for HF development in indicated age groups according to CART analysis. Patients were stratified according to the presence (y) or absence (n) of the corresponding risk factors. The number (N) of all patients and those who developed HF after starting the administration of anticancer agents (HF) are shown. The combinatorial risk, assessed by the relative hazard ratio (RHR), was expressed relative to the lowest hazard ratio in each age group. The combinatorial risk was stratified and color-coded into low- (1 ≤ RHR < 2, yellow), medium- (2 ≤ RHR < 3, orange), and high-risk (3 ≤ RHR, red) groups. The rightmost panels indicate RHR (red bars) and 95% confidence intervals (blue bars). *P < 0.05 and ***P < 0.001 compared to the lowest risk group (RHR = 1) in each age group

**Fig. 4:**
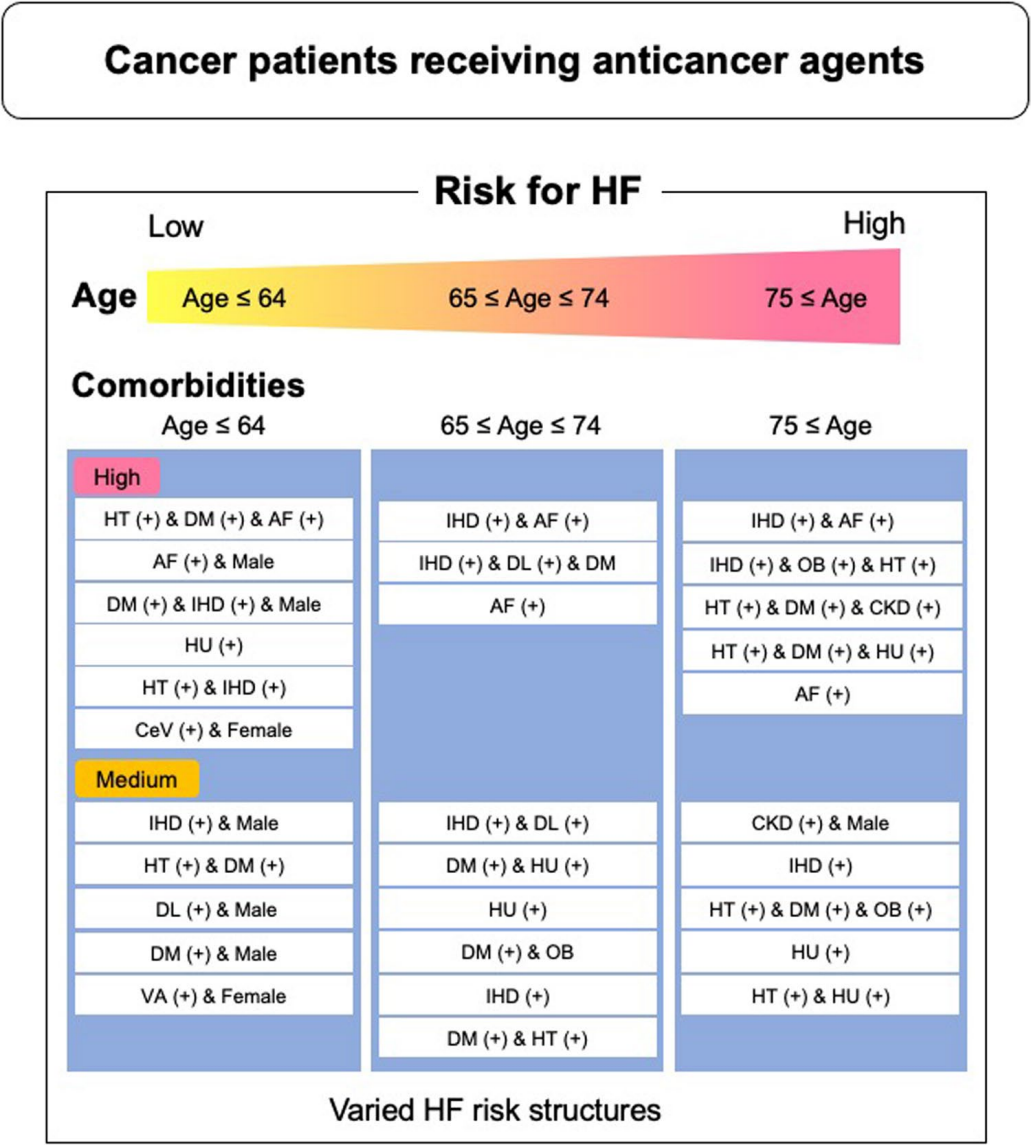
Graphical summary of risk structures for HF development. The risk factors for HF after treatment with anticancer agents formed context-dependent structures that were distinct among different age groups

We evaluated the risk factors of HF after treatment with anticancer agents by multivariable analysis. We investigated the impact of asymmetrical combinations of risk factors on HF in each of the three age groups and survival tree analyses based on the CART [15]. As the risk of developing HF depends on age (Fig. 2), we evaluated the structured risk factors with age stratification using the CART method (Fig. 3). Notably, the decision trees obtained by the CART method indicated that the risk factors for developing HF varied in each age group. IHD was the root node in the two older groups (Fig. 3). To better characterize the risk structures in the three age groups, we stratified the risk of HF development into three groups: low-risk (relative HR < 2 compared to the lowest HR within the corresponding age group), medium-risk (2 ≤ relative HR < 3), and high-risk (relative HR ≥ 3) (Fig. 3). In the group aged  ≤ 64 years, the combinations of “HT, DM, and AF,” “AF and male sex,” and “DM, IHD and male sex” were associated with high risk. In the two older groups, IHD was the key factor associated with high risk. However, in the group aged  ≥ 75 years, the combination of HT, DM, and CKD or hyperuricemia (HU) were also associated with high risk in the absence of IHD (Fig. 4). AF appeared in all three high-risk groups, and AF alone was sufficient to constitute a high-risk group. In this stratification of the three risk groups, each of the risk groups was constituted by various combinations of risk factors that were different among the age groups.

Next, we performed a subgroup analysis of the top six cancer types with the highest numbers of patients to clarify risk factors common to all cancer types. IHD and AF were significantly associated with HF development in all cancer types (Table 3). In addition, the results for IHD and AF were similar to those from the CART analysis of all patients and belonged to the high-risk group.

**Table 3:** Multivariate analysis for HF after treatment with anticancer agents in each cancer groups

Cancer type	Breast cancer	Prostate cancer	Colon cancer	Lung cancer	Hepatocellular carcinoma	Gastric cancer
Number	22,762	21,255	15,352	13,515	9597	9192
HF patients	438 (1.9%)	649 (3.1%)	511 (3.3%)	616 (4.6%)	496 (5.2%)	366 (4.0%)
Age						
Age ≤ 64 (reference)	1.00	1.00	1.00	1.00	1.00	1.00
65 ≤ Age ≤ 74	0.97 (0.70–1.35)	2.94 (1.47–5.86)**	1.25 (0.90–1.72)	1.21 (0.91–1.61)	1.38 (0.97–1.97)	0.87 (0.60–1.25)
75 ≤ Age	1.84 (1.34–2.53)	4.50 (2.28–8.86)**	2.26 (1.65–3.10)**	1.71 (1.27–2.30)**	1.52 (1.07–2.17)*	1.19 (0.83–1.70)
Male	1.99 (0.78–5.07)	–	1.12 (0.88–1.43)	1.21 (0.96–1.54)	0.77 (0.60–0.98)*	0.90 (0.67–1.23)
Obesity	1.31 (0.90–1.91)	1.09 (0.77–1.53)	1.45 (1.03–2.04)*	1.57 (1.21–2.04)**	1.26 (0.98–1.62)	0.94 (0.52–1.71)
Hypertension	1.25 (0.91–1.72)	1.67 (1.32–2.12)**	1.51 (1.16–1.95)**	1.04 (0.83–1.31)	1.10 (0.85–1.41)	0.95 (0.70–1.29)
Ischemic heart disease	1.87 (1.27–2.75)**	2.17 (1.69–2.78)**	1.60 (1.20–2.14)**	1.40 (1.06–1.85)*	1.51 (1.10–2.07)*	1.91 (1.38–2.66)**
Atrial fibrillation/flutter	2.72 (1.64–4.52)**	2.01 (1.44–2.79)**	1.98 (1.35–2.91)**	1.54 (1.06–2.23)*	1.65 (1.01–2.67)*	2.00 (1.22–3.27)**
Ventricular Arrhythmias	1.07 (0.50–2.30)	0.99 (0.55–1.80)	1.09 (0.57–2.07)	1.03 (0.48–2.20)	0.90 (0.33–2.42)	0.96 (0.40–2.31)
Diabetes mellitus	1.19 (0.87–1.64)	1.29 (1.03–1.63)*	1.21 (0.95–1.56)	1.17 (0.93–1.47)	1.13 (0.89–1.44)	0.95 (0.70–1.28)
Dyslipidemia	1.09 (0.77–1.53)	1.10 (0.84–1.43)	1.05 (0.80–1.39)	1.10 (0.85–1.43)	1.23 (0.90–1.67)	1.57 (1.13–2.19)**
Hyperuricemia	1.23 (0.51–2.93)	1.11 (0.78–1.57)	1.28 (0.81–2.01)	1.25 (0.84–1.87)	0.80 (0.51–1.27)	1.45 (0.87–2.40)
Chronic kidney disease (Stage G3—4)	1.22 (0.46–3.18)	1.36 (0.86–2.16)	0.43 (0.13–1.39)	1.05 (0.50–2.20)	2.16 (1.25–3.73)**	1.70 (0.80–3.62)
Cerebrovascular disease	1.66 (1.00–2.74)	0.85 (0.6–1.21)	1.06 (0.70–1.62)	1.22 (0.80–1.84)	1.17 (0.75–1.84)	0.96 (0.58–1.59)

*HR (99%CI)* hazard ratio (99% confidence interval)

^**^ < 0.001

^*^ < 0.01

## Discussion

In the present study, we evaluated the prevalence and risk factors of HF in 140,327 patients with various cancers who were treated by anticancer agents in Japan using a comprehensive database. First, among patients treated for cancer, 4.0% experienced HF after anticancer therapy. Second, the presence of CVDs and their risk factors predicted HF development after treatment with anticancer agents, and the risk factors for HF formed context-dependent structures that were distinct among different age populations. The strength of our study was the large sample size used to show the prevalence and risk factors for HF after anticancer treatment.

### HF prevalence and prognosis in cancer

We found that the prevalence of HF was 4.0% in the study population, which was apparently higher than that in the general population in Japan over the age of 45 (approximately 1.6%) [16] and in the United States over the age of 65 (approximately 1.0%) [17]. The apparently higher prevalence of HF in patients with cancer than in the general population is consistent with a prior study that showed an HF prevalence of 7.06% after the diagnosis of various types of cancer [18]. This may be explained by several factors. First, because both cancer and HF predominantly affect older people, a skewed proportion of older people in the study population may have contributed to the higher prevalence of HF. Second, the administration of anticancer agents and associated medical interventions such as high-volume hydration may increase the risk of HF after the administration of anticancer agents. Third, invasive surgical treatments also increase the risk of HF.

### Risk stratification in our analysis

The risk factors for HF development in this study included cardiovascular comorbidities and their risk factors [19, 20], which was consistent with previous reports regarding the risk factors for HF in general [21–25] and in patients taking anticancer agents [8, 9, 26, 27]. Therefore, our findings suggest that patients with cancer benefit from careful monitoring for HF risk factors as well as early intervention. However, it is unknown whether all risk factors contribute equally to HF or which risk factors predominantly cause HF in patients with cancer. To address this problem, we performed a machine learning-based analysis of the structured risk factor for HF after administration of anticancer agents using the CART algorithm. Our analysis showed that the effect of a given risk factor depends on the context of other risk factors, as illustrated by the distinct decision trees among the three age groups in the CART analysis. This finding implies that better risk stratification for individual patients may be achieved when combinations of risk factor structure are considered. In particular, IHD and AF form the roots of high-risk groups, and early detection and treatment of these diseases are considered important. As the DPC data or other EHR data are continuously generated in day-to-day clinical practice, better risk stratification may be achieved as the size of the dataset and knowledge grow. Such risk stratification is essential for the realization of precision medicine. Notably, the varied and context-dependent risk structures revealed in this study would not be suitable for a simple and static scoring system. However, the EHR has several limitations in assessing various aspects of patient status in clinical practice. It is crucial to carefully design how EHRs can be converted into datasets that are suitable for analyses. Therefore, the optimization of data extraction is essential for obtaining clinically meaningful findings from large datasets, such as the DPC database. Careful and iterative design is required to extract appropriate datasets for machine learning of the structured risk factors, and a collaborative framework among clinicians, clinical scientists, data scientists, and bioinformaticians is important.

### Study limitations

Several limitations of this study should be noted, including the nature of the DPC data structure and content. First, this was a retrospective study. DPC is a hospital-based, but not a patient-based, database that is primarily for recording medical procedures and costs but not clinical signs or laboratory data. Furthermore, medical records could not be retrieved when patients moved from DPC institutions to other DPC or non-DPC institutions. Second, diagnoses in the DPC database were less defined or validated than those in retrospective patient record-based or prospective registry studies. Third, we enrolled only those patients treated with anticancer agents. Therefore, a comparison between patients treated with and without anticancer agents cannot be made to evaluate the true impact of a given anticancer agent. Fourth, in this retrospective study, we summarized the big data and determined the structured risk factors of HF after treatment with anticancer agents but did not perform prognostic prediction or validation studies. These are required to confirm whether an intervention to control HF risk factors can reduce the risk of HF development and improve the prognosis of patients treated for cancer in the near future. Fifth, we have no data regarding stage of cancer, which can be directly related to prognosis.

## Conclusion

Using a comprehensive DPC database, the present study demonstrated that 4.0% of patients with cancer had HF after treatment with anticancer agents. The machine learning-based approach was able to develop complicated HF risk structures for these patients after age stratification. The findings obtained in the studies such as this one are essential to achieve precision medicine for better outcomes for patients with cancer.

## Supplementary Information

Below is the link to the electronic supplementary material.

**Supplementary file 1: (DOCX 18KB)**

## Data Availability

The data presented in this study are available on request from the corresponding author with the permission of Medical Data Vision Co., Ltd.

## Abbreviations

AF: Atrial fibrillation
CART: Classification and regression tree
CVDs: Cardiovascular diseases
DPC: Diagnosis procedure combination
EHR: Electronic health record
HF: Heart failure
ICD-10: International classification of disease, 10th revision
IHD: Ischemic heart disease
