# Supplementary material for: Risk factor structure of heart failure in patients with cancer after treatment with anticancer agents’ assessment by big data from a Japanese electronic health record
Source: Heart Vessels. 2023 Jan 27;38(6):793–802. doi: 10.1007/s00380-023-02238-9 (PMC10182140; doi:10.1007/s00380-023-02238-9)
Supplement: Supplementary file 1 — (DOCX 18KB) [file 380_2023_2238_MOESM1_ESM.docx]

**Supplementary Table 1. Inclusion and exclusion criteria**

| **Inclusion criteria to enroll patients**  ・First diagnosis of cancer between Apr, 2008 – Jan, 2017  ・Anticancer agents administration after diagnosis of cancer between Apr, 2008 – Jan, 2017  ・Age ≥ 18 years old |
| --- |
| **Exclusion criteria to enroll patients**  ・First diagnosis of cancer before Apr, 2008  ・Not detected of anticancer agents between Apr, 2008 – Jan, 2017  ・Age < 18 years old |

**Supplementary Table 2. ICD-10 code for heart failure diagnosis and comorbidies in this study.**

| **Disease name** | **ICD-10 code** |
| --- | --- |
| Heart failure  Hypertension  Ischemic heart disease  Atrial fibrillation/flutter  Ventricular Arrhythmias  Diabetes mellitus  Dyslipidemia  Hyperuricemia  Chronic kidney disease  Severe renal failure  Cerebrovascular disease  Acute myocardial infarction  Cardiopulmonary arrest  Acute respiratory distress syndrome  Severe pneumonia  Pleuritis | I50.0, I50.1, I50.9, I11.0, I42.7  I10, I15.9  I20, I25  I48  I47.0, I47.2, I49.0, I49.3  E10-14  E78  E79  N18.3, N18.4, N18.9, N18.5, N19  N17, N18.5  I61, I63, I64  I21-24  I46, R96  J80  J10.0, J11.0, J12-18, J69  A15.6, A16.5, R09.1, J90, J91, J94 |

**Supplementary Table 3. Heart failure definition**

| **Inclusion criteria for HF**  ・ICD-10 codes [I50.0, I50.1, I50.9, I11.0, I42.7]  ・Use of one or more therapeutic drugs for HF after HF diagnosis |
| --- |
| **Exclusion criteria for HF**  ・Diagnosis of acute myocardial infarction on the same day as HF  ・Diagnosis of other conditions indistinguishable from HF on the same day as HF |
